# Effects of Rare Microbiome Taxa Filtering on Statistical Analysis

**DOI:** 10.3389/fmicb.2020.607325

**Published:** 2021-01-12

**Authors:** Quy Cao, Xinxin Sun, Karun Rajesh, Naga Chalasani, Kayla Gelow, Barry Katz, Vijay H. Shah, Arun J. Sanyal, Ekaterina Smirnova

**Affiliations:** ^1^Department of Biostatistics, Epidemiology and Informatics, Perelman School of Medicine, University of Pennsylvania, Pennsylvania, PA, United States; ^2^Biostatistics Department, Virginia Commonwealth University, Richmond, VA, United States; ^3^Bioinformatics Department, Virginia Commonwealth University, Richmond, VA, United States; ^4^Department of Biostatistics, Harvard University, Boston, MA, United States; ^5^Division of Gastroenterology, Department of Internal Medicine, Indiana University, Indianapolis, IN, United States; ^6^Department of Biostatistics, Indiana University, Indianapolis, IN, United States; ^7^Division of Gastroenterology, Department of Internal Medicine, Mayo Clinic, Rochester, MA, United States; ^8^Division of Gastroenterology, Hepatology and Nutrition, Department of Internal Medicine, Virginia Commonwealth University, Richmond, VA, United States

**Keywords:** filtering, fast permutation test, quality control, microbiome, contaminants

## Abstract

**Background:** The accuracy of microbial community detection in 16S rRNA marker-gene and metagenomic studies suffers from contamination and sequencing errors that lead to either falsely identifying microbial taxa that were not in the sample or misclassifying the taxa of DNA fragment reads. Removing contaminants and filtering rare features are two common approaches to deal with this problem. While contaminant detection methods use auxiliary sequencing process information to identify known contaminants, filtering methods remove taxa that are present in a small number of samples and have small counts in the samples where they are observed. The latter approach reduces the extreme sparsity of microbiome data and has been shown to correctly remove contaminant taxa in cultured “mock” datasets, where the true taxa compositions are known. Although filtering is frequently used, careful evaluation of its effect on the data analysis and scientific conclusions remains unreported. Here, we assess the effect of filtering on the alpha and beta diversity estimation as well as its impact on identifying taxa that discriminate between disease states.

**Results:** The effect of filtering on microbiome data analysis is illustrated on four datasets: two mock quality control datasets where the same cultured samples with known microbial composition are processed at different labs and two disease study datasets. Results show that in microbiome quality control datasets, filtering reduces the magnitude of differences in alpha diversity and alleviates technical variability between labs while preserving the between samples similarity (beta diversity). In the disease study datasets, DESeq2 and linear discriminant analysis Effect Size (LEfSe) methods were used to identify taxa that are differentially abundant across groups of samples, and random forest models were used to rank features with the largest contribution toward disease classification. Results reveal that filtering retains significant taxa and preserves the model classification ability measured by the area under the receiver operating characteristic curve (AUC). The comparison between the filtering and the contaminant removal method shows that they have complementary effects and are advised to be used in conjunction.

**Conclusions:** Filtering reduces the complexity of microbiome data while preserving their integrity in downstream analysis. This leads to mitigation of the classification methods' sensitivity and reduction of technical variability, allowing researchers to generate more reproducible and comparable results in microbiome data analysis.

## 1. Introduction

Studies of microbiota association and human disease states have received increasing attention over the last decade (Nguyen et al., 2015). It was shown that microbiota composition plays an important role in the development of multiple diseases, including inflammatory bowel disease (Huttenhower et al., 2014), diabetes (Proctor, 2014; Pascale et al., 2019), preterm birth (DiGiulio et al., 2015; Callahan et al., 2017), and liver diseases (Puri et al., 2018; Smirnova et al., 2020). Next-generation sequencing (NGS) of the 16S rRNA marker is currently among the most widely used methods for microbial organism identification. In these studies, samples collected at different body sites (e.g., vaginal swab, stool, or blood) give counts of DNA fragments, which are then grouped into similar microbial organisms, usually referred to as taxa. Hence, the resulting data is usually referred to as the “taxa table” or “derived feature data.” In contrast to other -omics measurements, microbiome data are very sparse as many taxa are rare and often have zero counts in most samples.

The extreme levels of sparsity in microbiome datasets are one of the major challenges in data analysis. Indeed, it is not unusual to have over 90% of 0s in this data, as it contains a large number of rare taxa observed in as few as 1 to 5% of samples. Recent microbiome quality control studies indicate that many rare taxa are caused by sequencing artifacts (Lahr and Katz, 2009), contamination, and/or sequencing errors (Knights et al., 2011; Ravel et al., 2011; Fettweis et al., 2012; Sinha et al., 2015; Callahan et al., 2016). Common bioinformatics pipelines that derive taxonomic features tables, such as QIIME (Caporaso et al., 2010) and dada2 (Callahan et al., 2016), implement a number of quality control and potential contaminant removal functions. These functions trim sequences to a specified length, remove sequences shorter than that length, and filter based on several ambiguous bases, a minimum quality score, and the expected errors in a read. Dada2 also, by default, performs low-level filtering by removing singletons while considering each sample individually. Yet, many rare and low-prevalence features, as well as contaminants, may still remain (Davis et al., 2018). The most common approach to address this problem in the derived feature data, at the same time increasing the power of subsequent statistical testing by reducing the number of multiple hypotheses, is filtering: removing spurious taxa from the 16S data set. Most filtering approaches are based on the rules of thumb, which vary from lab-to-lab. Such approaches are implemented in R packages genefilter (Gentleman et al., 2020) and phyloseq (McMurdie and Holmes, 2013), as well an in QIIME bioinformatics pipeline function filter_otus_from_otu_table.py (Caporaso et al., 2010). Recently, a filtering loss measure and a principled filtering test, namely PERFect (Smirnova et al., 2019), was introduced for deciding which taxa to remove. These methods are implemented in Bioconductor package PERFect (Smirnova and Cao, 2020), which includes a novel fast implementation of the permutation PERFect method. The implemented approach successfully reduces the original algorithm running time by almost four times.

While some techniques have been proposed to detect and remove contaminant and/or rare taxa, the literature in this research area is relatively scarce. Davis et al. (2018) addressed this problem by introducing the R package decontam that identifies contaminants by: (1) inversely correlating taxa frequencies with sample DNA concentration and (2) using the prevalence of sequenced negative controls (Salter et al., 2014). This method requires the auxiliary data from DNA quantitation, which is in most cases intrinsic to sample preparation, or negative controls data that is intrinsic to the sequencing protocol. This approach is closely related but not identical to filtering.

Traditional filtering methods were previously compared to the PERFect approach proposed by Smirnova et al. (2019) and tested on two datasets acquired from mock community experiments carried out at Virginia Commonwealth University (VCU) (Fettweis et al., 2012; Brooks et al., 2015) and a reagent and laboratory contamination dataset (Salter et al., 2014). The authors used the number of contaminant taxa removed from the mock datasets as the method evaluation criteria. However, in practice, filtering is used as an intermediary step applied to the derived taxonomic feature table prior to data analysis. While filtering is a commonly used and recommended approach (Goodrich et al., 2014; Cullen et al., 2020), its benefits on data analysis and the effects on the scientific conclusions drawn from filtered and unfiltered data have not been reported.

The objectives of the current study are to evaluate: (1) the effects of filtering on technical variability for identical mock samples processed under different conditions; (2) the advantages and disadvantages of using filtering for detecting significant taxa discriminating two groups of medical conditions. To address the first goal, we analyze the recent MicroBiome Quality Control (MBQC) project (Sinha et al., 2015) that includes 1, 016 oral mock samples sequenced at 15 laboratories and the results were then randomly distributed to 9 bioinformatics facilities for taxonomic classification; and the previously studied laboratory contamination dataset (denoted as Salter data) (Salter et al., 2014). To address the second goal, we analyze two novel datasets on the gut microbiome studies on the TREAT consortium alcoholic hepatitis study (Smirnova et al., 2020) and Human Microbiome Project inflammatory bowel disease (Lloyd-Price et al., 2019). To evaluate the effects of filtering, we concentrate on (1) alpha (within) and beta (between) samples diversity analysis and (2) identification of significant taxa using random forest classification, LEfSe and DESeq2 methods. Finally, we discuss the filtering and contaminant removal methodologies and show that these approaches have complementary effects.

## 2. Motivating Datasets

### 2.1. Mock Data

#### 2.1.1. The Microbiome Quality Control Data

Consider the dataset from the MBQC project, a collaborative effort designed to comprehensively evaluate sample processing and computational methods for human microbiome data analysis (Sinha et al., 2015). There were four types of samples in the full dataset: (1) 11 unique fresh stool samples; (2) seven unique freeze-dried stool samples; (3) two unique chemostat samples generated from a Robogut; and (4) two artificial colonies representing the gut and oral cavity. The aliquot of these samples was first randomly sequenced at 15 laboratories, and the results were then randomly distributed to 9 bioinformatics facilities for taxonomic classification. Each bioinformatics facility followed an in-house analysis pipeline to generate the final feature table (Sinha et al., 2015); protocol details can be obtained from the MBQC project website (MBQC, 2015). Here, we considered the oral artificial communities data comprised of 22 true taxa. The MBQC project identified a total of 27, 140 taxa across the four types of samples. For this analysis, 14, 861 taxa identified in non-oral artificial community samples (i.e., fresh stool, freeze-dried stool, chemostat, and gut artificial colony) were excluded; 1, 277 taxa that matched names at the species level were combined; finally, 10, 210 taxa that appeared in less than 5% of the samples were removed. The final dataset considered for this analysis contained 1, 016 samples and 792 taxa. A limitation of this dataset is that the samples were created from the species in prescribed proportions; however, after the samples were processed many taxa were only identified up to the genus level (higher-order phylogenetic hierarchy). As a consequence, only two signal taxa, *Veillonellaceae Veillonella Parvula* and *Coriobacteriaceae Eggerthella Lenta* were correctly detected while the other 20 signal species are among the 184 taxa identified at the genus level.

**Figure 1:**
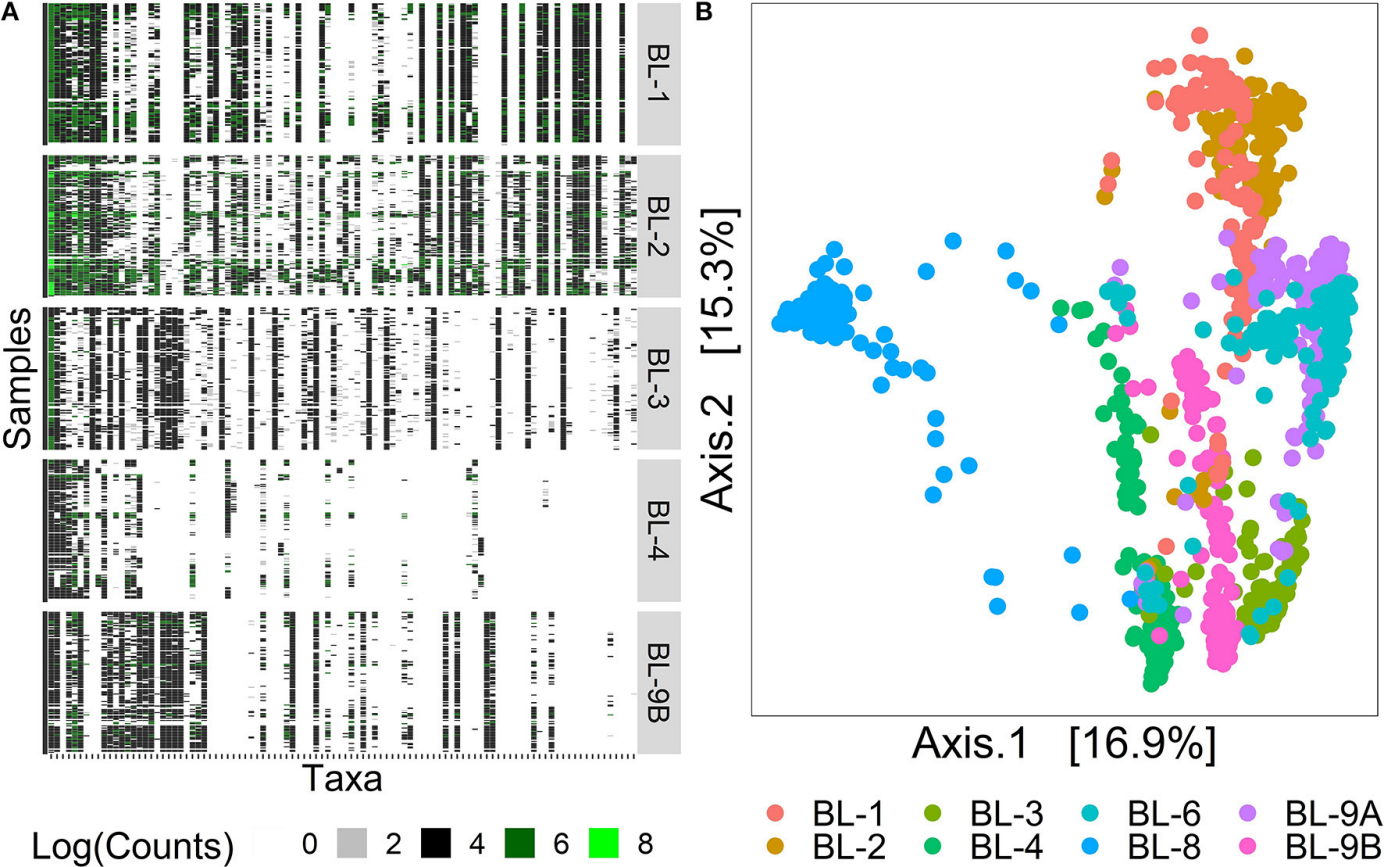
Heat map and PCoA plot of MBQC data. **(A)** The heat map of 100 observed taxa on the log-scale, with taxa on the *x*-axis arranged in decreasing abundance order and samples on the *y*-axis arranged by processing institutes. **(B)** The PCoA plot of 1016 samples, colored by the processing institutes. Data source: (Sinha et al., 2015).

Figure 1A displays the log-counts heat map for the 100 most abundant taxa for the first five labs, arranged in decreasing order of abundance. Here, we rank taxa abundance by the number of samples a taxon is present in, where the most abundant taxon is ranked as 1, the second most abundant as 2, and so on. The white areas of the heatmap in the lower right corner indicate unobserved taxa, showing the decrease of signal strength with different processing institutes/labs. Figure 1B displays the Bray–Curtis distance (Quaak and Kuiper, 2011) principal coordinates analysis (PCoA) plots for 1, 016 samples from the heat map on the left. The first two axes that explain 32.2% of variability are shown on the plot. The samples were clustered by bioinformatics labs, indicating differences across samples processed at different institutes even though they contain the same signal species. These observations highlight the strong effects of the sequencing and bioinformatics protocol choice on taxa identification. Filtering, which removes rare taxa displayed in columns on the right-hand side of the heatmap in Figure 1A is one approach that could mitigate these differences. Left unresolved, this problem may cause a number of practical issues including (1) falsely inflating within-sample diversity, called alpha diversity (Park and Allaby, 2017); (2) obscuring true distances between samples, called beta diversity (Park and Allaby, 2017); and (3) interpreting rare taxa as disease biomarkers (especially in low sample biomass environments).

#### 2.1.2. The Reagent and Laboratory Contamination Data

**Figure 2:**
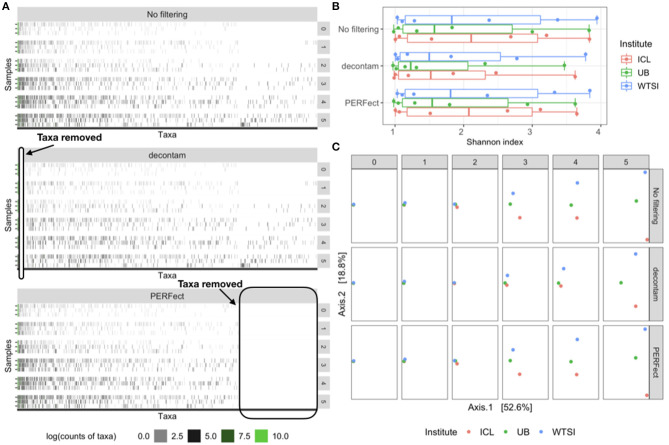
Comparison of the original data (no filtering), contaminant removal (decontam frequency) and filtering (PERFect simultaneous) methods. **(A)** Heatmap of log-transformed taxa counts in decreasing abundance order on the x-axis and samples by dilution level on the y-axis for the original data (top panel), data where contaminants are removed using decontam (middle panel) and rare taxa filtered using PERFect (bottom panel). True taxa are colored in green to the left of each heatmap; ovals indicate taxa removed by decontam and PERFect methods. **(B)** Alpha diversity for the three comparisons colored by dilution level and processing institute. **(C)** Beta diversity Bray–Curtis distances plots colored by processing institute and arranged by dilution level (rows) and three taxa removal methods (columns). Data source: (Salter et al., 2014).

The reagent and laboratory contamination study was designed to determine the effects of DNA extraction kits and other laboratory reagent contamination on sequencing output (Salter et al., 2014). These data contain mock samples of a pure *Salmonella bongori* culture that had been processed at three different institutes: (1) Imperial College London (ICL); (2) University of Birmingham (UB); and (3) Wellcome Trust Sanger Institute (WTSI). Each mock sample underwent five rounds of serial 10-fold dilutions to generate a series of high (dilution = 0) to low (dilution = 5) biomass samples. The amplicon sequencing data was processed using the R package dada2 to generate a table of exact amplicon sequence variants (ASV); processing steps details are described in the R markdown script salter_metagenomics.Rmd Callahan (2018). The data visualization heatmap in Figure 2A top panel displays the log-counts heatmap for 635 observed taxa generated using 40 Polymerase Chain Reaction (PCR) cycles. The taxa on the horizontal axis are arranged in decreasing order of abundance and the 18 samples on the vertical axis arranged by high to low (0–5) degrees of dilution. Results indicate that as the dilution number increases, true taxa contain fewer signals and are observed in lower counts, which makes it difficult to separate the signal (i.e., true features of *Salmonella bongori* culture in the mock samples) from the noise (i.e., features derived in the taxa table as a part of sequencing, amplification, or experimental error).

### 2.2. Disease Study Data

In many microbiome studies, it is of interest to identify specific bacterial taxa that discriminate between two or more disease groups. We investigate the effects of filtering on identifying specific taxa that contribute to these differences using two recently reported microbiome studies.

#### 2.2.1. Alcoholic Hepatitis Data

The study to characterize changes in the fecal microbiome due to alcohol consumption and alcoholic hepatitis was performed by the sites involved in the TREAT consortium from 2014–2018 (Smirnova et al., 2020). A total of 78 participants [healthy control (HC), *n* = 24; heavy drinking control (HDC), *n* = 20; moderate alcoholic hepatitis (MAH), *n* = 10; severe alcoholic hepatitis (SAH), *n* = 24] were studied. To interrogate and characterize gut microbiome composition, the 16S data was used. Length Heterogeneity PCR (LH-PCR) fingerprinting was routinely used to rapidly survey the samples and standardize the community amplification. The microbial taxa associated with the gut mucosal microbiome were then interrogated using Multitag Sequencing (MTS) on the samples (Gillevet et al., 2010). The operational taxonomic unit (OTU) table was obtained using customized PERL scripts as described in the Smirnova et al. (2020) supporting information. Results indicated that in random forest classification models, alcoholic hepatitis (moderate and severe alcoholic hepatitis groups combined; *n* = 34) was associated with a distinct microbiome signature compared to heavy drinking controls (AUC = 0.826), and multiple microbial genera were identified as the key contributors to these differences.

#### 2.2.2. Inflammatory Bowel Disease Data

The inflammatory bowel disease (IBD) data were generated as a part of the NIH Common Fund's Integrative Human Microbiome Project (iHMP/HMP2). The initial findings and multi-omic datasets from these studies were published in the Nature family of journals in May and June of 2019 (Lloyd-Price et al., 2019), and 16S data are publicly available through the HMP Data Coordination Center (HMP-DACC) and HMP2Data Bioconductor package (Stansfield et al., 2020) in R and processing steps are described in Lloyd-Price et al. (2019) and NIH Human Microbiome Project (2015). The subset of 132 patients [control (non-IBD), *n* = 46; Crohn's disease (CD), *n* = 86] with the open-source 16S data available through the HMP2Data package was selected for the analyses presented in this manuscript.

**Figure 3:**
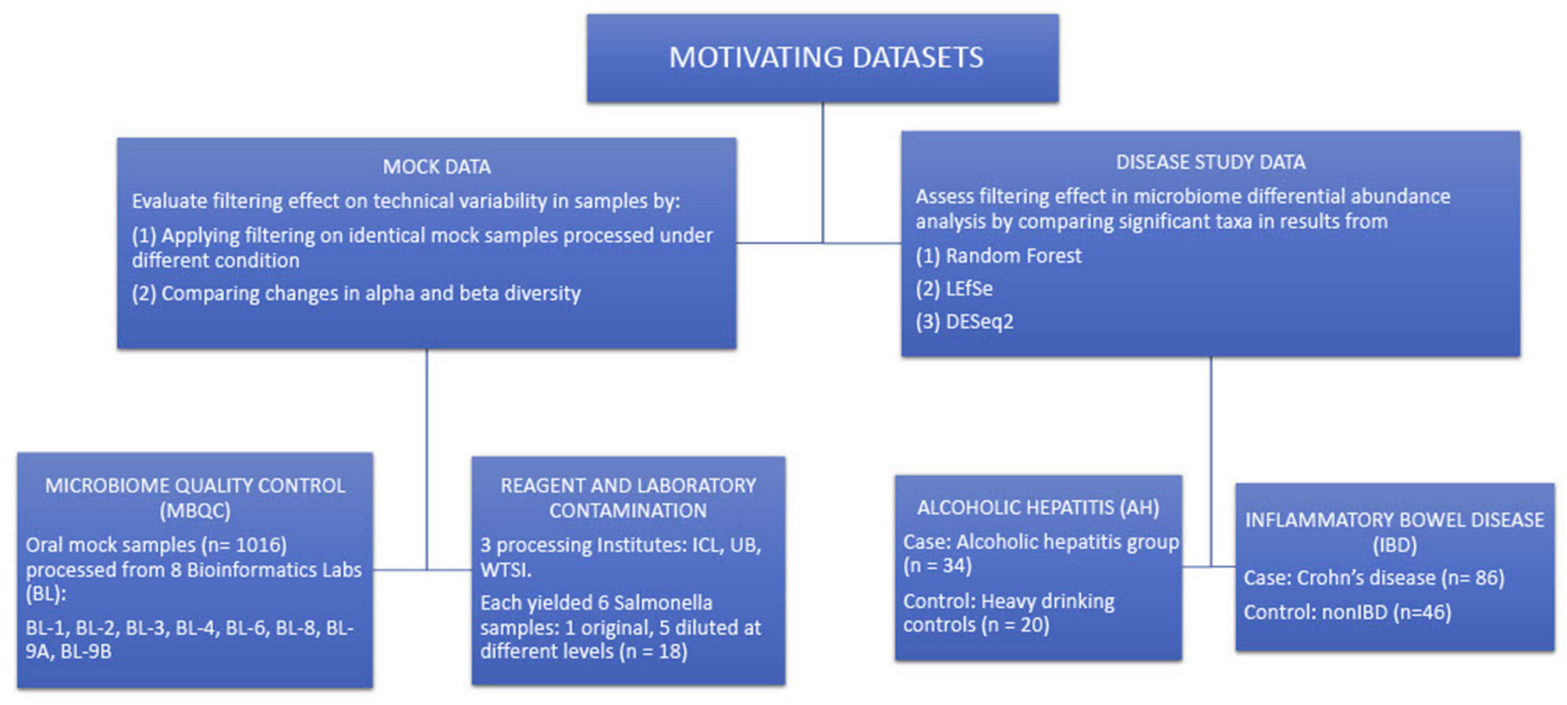
Summary of the structure of the four motivating datasets and types of analyses performed on each dataset.

Figure 3 summarizes the structure of the four motivating datasets and analyses applied to each dataset. Briefly, alpha and beta diversity analyses were applied to two mock data sets to illustrate the effects of filtering on reducing technical variability between different processing techniques. LEfSe, DESeq2, and random forest methods were applied to the disease study datasets to compare the differences in detecting differentially abundant taxa in a pairwise comparison of cases and controls.

## 3. Methods

### 3.1. Filtering Methods

Currently, there are only a few statistically motivated filtering methods used to alleviate the issue of contaminant and rare taxa. The majority of currently used filtering methods are based on a heuristic non-statistical rule, with two methods where a threshold is derived statistically from the data. Here, we give a brief overview of these methods.

#### 3.1.1. Rule of Thumb Approaches

In practice, filtering is a variation of an *ad-hoc*, albeit simple, procedure. One of the most widely used techniques for filtering in microbiome studies selects taxa that have several counts above *m*=0 in at least *k* samples. This approach is borrowed from the RNA-seq gene expression literature and is implemented in the R package genefilter (Gentleman et al., 2020) and in QIIME bioinformatics pipeline function filter_otus_from_otu_table.py (Caporaso et al., 2010). The choice of the threshold *k* comes from the count that is 0.1% of the minimal library size (the total number of count reads in the sample). For example, often the minimal library size is set to 5, 000 reads, and popular filtering rules thus keep taxa present in at least *k*=5 samples. Another popular approach is to remove taxa that are observed in fewer than *k*% of the samples. The advantage of these methods is that they are simple, intuitive, and easy to communicate with collaborators. However, they do not have an explicit loss function and objective criteria for choosing the tuning parameters *m* and *k*.

#### 3.1.2. Statistical Threshold Selection

In contrast to the rule of thumb approaches where thresholds for filtering taxa are determined heuristically, the statistical approach selects an empirical filtering threshold based on the information given by the data. It extends the traditional rule of thumb filtering approaches to find the best subset of retained taxa for further analysis by implementing statistical data-driven significance cut-off thresholds. The current method for such an approach is PERFect, a principled filtering test that removes taxa with an insignificant contribution to the total covariance (Smirnova et al., 2019). Specifically, this method derives a statistical threshold for separating the signal (features with a strong contribution to the taxa table) from noise (rare features derived in the taxa table as a part of sequencing, amplification, or experimental error) taxa. The threshold is derived based on the dramatic increases in the loss due to filtering the set of potential noise features and quantifying the chance that this increase in the loss due to taxa filtering is due to randomness using permutation tests. PERFect introduces two filtering algorithms, namely, PERFect simultaneous and PERFect permutation. The former algorithm assumes that a large percentage of the taxa has a low signal and the difference in filtering loss for all taxa are fit using one distribution, whereas the latter algorithm fits a distribution for each set of filtered taxa. One drawback of the permutation filtering method is that it might be computationally expensive. Indeed, given that *k* permutations are performed for each taxon *j*=2, 3, …, *p*, the algorithm requires a total of *k*(*p* − 1) permutations, where *k* and *p* are large. Thus, the newer version of this package (see Supplementary Information), employs parallel processing and an unbalanced binary search algorithm (Morin, 2014) that optimally finds the cut-off taxon *j* to remove the set of taxa without building the permutation distribution and computing the *p*-values for all *p* − 1 taxa.

### 3.2. Contaminant Removal Method

Contaminants in microbiome studies may arise from external sources such as the body of the study participant or sample collector (Kitchin et al., 1990; Meadow et al., 2015), sample collection instruments and laboratory reagents (Salter et al., 2014; Jousselin et al., 2015; Glassing et al., 2016) or from internal sources (cross-contamination) when samples were mixed with each other during sample processing (Jousselin et al., 2015) or sequencing (Larsson et al., 2018). The recently developed contaminants removal method Decontam (Davis et al., 2018) identifies external contaminants by either (1) inversely correlating taxa frequencies with sample DNA concentration or (2) by using the prevalence of sequenced negative controls. Our results suggest that Decontam removes abundant taxa that are likely contaminants but does not address the issue of rare taxa. A practical limitation of this method is that it requires auxiliary information from DNA quantitation or negative controls that is intrinsic to the sequencing protocol and might not always be available.

### 3.3. Statistical Analysis Methods

Statistical analyses were performed in R 3.6.0. Within sample (alpha) (Park and Allaby, 2017) and between samples (beta) (Park and Allaby, 2017) diversity were used to evaluate the effects of filtering on reducing technical variability in mock datasets. Differences in estimated alpha diversity between processing labs were evaluated using Dunn's test with Benjamini–Hochberg controlling the false discovery rate (Benjamini and Hochberg, 1995) multiple comparisons adjustment. Principal coordinates analysis was performed using Bray–Curtis distances to visually display variability between samples. Axes variances were calculated without zeroing negative eigenvalues.

A number of methods for disease state prediction and differentially abundant taxa identification commonly used in metagenomic data analysis are considered in studying the effect of filtering. These methods were first performed on unfiltered and filtered data, then features importance for prediction and differentially abundant taxa selected by each method were compared. For predictive modeling, random forest (Breiman, 2001), which is extensively applied in computational biology and genomics (Statnikov et al., 2013), was used to identify the set of most predictive taxa based on their Mean Decrease Gini measures. The classification model diagnostic ability in filtered and unfiltered models was compared using the area under the receiver operating characteristic curve (AUC).

To identify differentially abundant taxa, DESeq2 (Love et al., 2014) and linear discriminant analysis effect size (LEfSe) (Segata et al., 2011) were used. DESeq2 fits a negative binomial generalized linear model for each taxon count to obtain estimates of a log-fold change between two classes and performs a Wald test on this value for significance testing. A detailed guide and standard workflow for DESeq2 can be found in the package's vignette (Love et al., 2020). LEfSe determines differentially abundant features by pairing non-parametric standard tests for statistical significance with linear discriminant analysis (LDA), allowing researchers to further identify features that are consistent with biologically meaningful categories (Segata et al., 2011). However, there are some limitations to these methods when applied to microbiome data. Our results suggest that in many instances each of these three methods tends to flag a taxon as significant when the difference between two classes is driven by outlier counts. For example, a rare taxon that is absent for most samples and present in a few samples of one class can potentially be classified as a differentially abundant taxon.

## 4. Results and Discussion

PERFect simultaneous and permutation filtering approaches were previously validated (Smirnova et al., 2019) on three mock community data sets (Knights et al., 2011; Ravel et al., 2011; Fettweis et al., 2012) using the number of contaminant taxa correctly removed as an efficiency criterion. Here, we concentrate on the effects of filtering on downstream analyses, using the two major exploratory analyses used in microbiome research, alpha and beta diversity, as well as its impact on identifying taxa that discriminate between disease states.

### 4.1. The MicroBiome Quality Control Data Analysis

**Figure 4:**
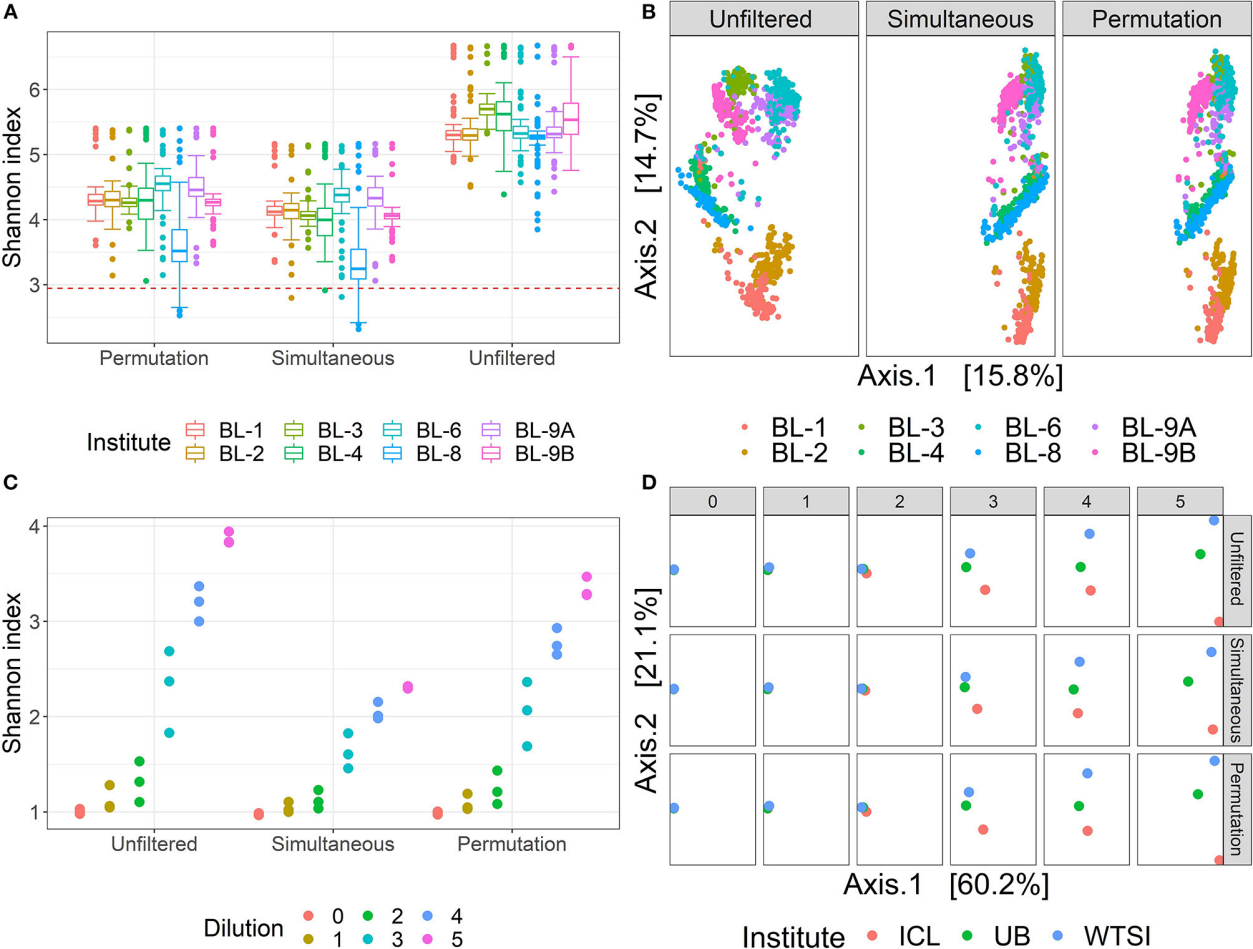
Diversity comparison on MBQC and Salter data. **(A)** Shannon index for the original data and two filtered data, colored by the bioinformatics labs. The horizontal dashed line represents the true Shannon index. **(B)** PCoA plots of the unfiltered, simultaneous, and permutation PERFect filtered data colored by bioinformatics processing institutes. Data source for (A) and (B): (Sinha et al., 2015). **(C)** Shannon index for the original data and two filtered data, colored by the dilution levels. **(D)** PCoA plots of the unfiltered and filtered data at different dilution levels, colored by the processing institutes. Data source for (C) and (D): (Salter et al., 2014).

One of the main goals of the MBQC project was to understand the major differences in technology and methods for analyzing human microbiome data. This was achieved by analyzing the observed taxa variation between (1) the labs that sequenced samples according to their internal protocol and (2) bioinformatics pipelines used to perform taxonomic classification. Here, we concentrate on the effect of bioinformatics processing laboratories on the observed oral mock community data measured by alpha and beta diversity, two of the most commonly used summaries in microbiome research. Figure 4A shows the Shannon index, the most widely-used diversity metric that accounts for both abundance and evenness of the species present (Reese and Dunn, 2018) in the unfiltered and filtered data. The Shannon index, *H* is defined as $H=-\overset{S}{\underset{i=1}{\sum }}p_{i}\ln\left(p_{i}\right)$, where *p*_*i*_ is the proportion of total sample represented by species *i* and *S* is the total number of species in the community. This plot and the summary statistics in Table 1 indicate a decrease in the Shannon index between the unfiltered and filtered data, implying a reduction in the diversity of taxa. Specifically, the diversity decreases from 792 taxa to 175 and 222 taxa using the simultaneous and permutation method respectively, while retaining 22 true taxa. Since both filtering methods remove more than 70% of taxa, the distribution of the remaining taxa shifts in favor of the true taxa by increasing their proportions in the samples, resulting in less even communities. As a result, this reduction of alpha diversity of samples tends toward the true alpha diversity as indicated by the red dashed line in Figure 4A.

**Table 1:** Summary statistics of the Shannon index for each processing lab. Data source: (Sinha et al., 2015).

		**BL-1**	**BL-2**	**BL-3**	**BL-4**	**BL-6**	**BL-8**	**BL-9A**	**BL-9B**
Median	Unfiltered	5.301	5.293	5.700	5.622	5.324	5.270	5.317	5.536
	Simultaneous	4.122	4.147	4.061	3.998	4.381	3.247	4.332	4.061
	Permutation	4.287	4.301	4.261	4.300	4.552	3.519	4.459	4.269
IQR	Unfiltered	0.137	0.175	0.165	0.446	0.181	0.062	0.161	0.477
	Simultaneous	0.135	0.231	0.123	0.420	0.205	0.456	0.280	0.083
	Permutation	0.166	0.235	0.127	0.475	0.224	0.489	0.288	0.098

To study the effect of filtering on differences across bioinformatics processing labs, we applied Dunn's test with a Benjamini–Hochberg correction for multiple testing to all possible pairwise Shannon alpha diversity comparisons between processing labs. Results are summarized in Table 2. Since all samples contained the same mock communities, in the absence of technical variability, none of the differences should be significant. For the unfiltered data, 21 out of 28 possible pairs have significant differences in alpha diversity at the 0.05 significance level. Applying simultaneous and permutation filtering decreases differences in alpha diversity for most pairs. Moreover, *there are a total of* 4 *and* 8 *pairwise comparisons that are no longer significant at the* 0.05 *level after simultaneous and permutation filtering was applied respectively*. While filtering does not remove all differences due to processing labs, these results indicate that it dramatically alleviates differences in alpha diversity estimates caused by the lab-to-lab variability.

**Table 2:** Pairwise comparisons of the Shannon index between laboratories using Dunn's test for each dataset. Data source: (Sinha et al., 2015).

	**Unfiltered**		**Simultaneous**		**Permutation**	
**Comparison**	**Difference**	***P*-values**	**Difference**	***P*-values**	**Difference**	***P*-values**
BL-1 - BL-2	0.00	0.4990	0.20	0.4214	−0.06	0.4778
BL-1 - BL-3	−10.75	<0.0001	2.52	0.0074	1.27	0.1307
BL-2 - BL-3	−10.83	<0.0001	2.35	0.0115	1.33	0.1283
BL-1 - BL-4	−7.22	<0.0001	3.90	0.0001	0.29	0.4173
BL-2 - BL-4	−7.28	<0.0001	3.73	0.0001	0.35	0.4088
BL-3 - BL-4	3.91	0.0001	1.25	0.1243	−1.01	0.1816
BL-1 - BL-6	−1.63	0.0632	−6.35	<0.0001	−7.10	<0.0001
BL-2 - BL-6	−1.64	0.0646	−6.60	<0.0001	−7.10	<0.0001
BL-3 - BL-6	9.36	<0.0001	−8.78	<0.0001	−8.23	<0.0001
BL-4 - BL-6	5.70	<0.0001	−10.47	<0.0001	−7.55	<0.0001
BL-1 - BL-8	2.47	0.0090	11.30	<0.0001	9.99	<0.0001
BL-2 - BL-8	2.49	0.0089	11.19	<0.0001	10.13	<0.0001
BL-3 - BL-8	13.27	<0.0001	8.51	<0.0001	8.50	<0.0001
BL-4 - BL-8	9.81	<0.0001	7.60	<0.0001	9.92	<0.0001
BL-6 - BL-8	4.16	<0.0001	17.93	<0.0001	17.36	<0.0001
BL-1 - BL-9A	−1.09	0.1535	−5.46	<0.0001	−5.74	<0.0001
BL-2 - BL-9A	−1.10	0.1583	−5.70	<0.0001	−5.73	<0.0001
BL-3 - BL-9A	9.66	<0.0001	−7.86	<0.0001	−6.88	<0.0001
BL-4 - BL-9A	6.09	<0.0001	−9.46	<0.0001	−6.14	<0.0001
BL-6 - BL-9A	0.51	0.3167	0.77	0.2455	1.24	0.1311
BL-8 - BL-9A	−3.57	0.0003	−16.78	<0.0001	−15.76	<0.0001
BL-1 - BL-9B	−6.44	<0.0001	3.32	0.0006	1.58	0.0840
BL-2 - BL-9B	−6.49	<0.0001	3.14	0.0011	1.65	0.0770
BL-3 - BL-9B	4.55	<0.0001	0.70	0.2609	0.27	0.4090
BL-4 - BL-9B	0.71	0.2556	−0.56	0.2996	1.33	0.1233
BL-6 - BL-9B	−4.92	<0.0001	9.79	<0.0001	8.79	<0.0001
BL-8 - BL-9B	−9.00	<0.0001	−8.06	<0.0001	−8.49	<0.0001
BL-9A - BL-9B	−5.32	<0.0001	8.82	<0.0001	7.37	<0.0001

To assess the effect of filtering on beta diversity, we calculated the pairwise Bray–Curtis distances between samples using a combined taxa matrix which consists of the unfiltered taxa matrix, and the taxa filtered matrices of PERFect simultaneous and PERFect permutation each at the *p*-value threshold of 0.1. The PCoA ordination plot for the first two axes which explain 30.5% of the variability in the data is shown in Figure 4B. Three filtering methods (unfiltered, simultaneous, and permutation PERFect) are arranged in columns, and samples are colored according to 8 processing institutes. Figure 4B shows that while data were clustered in a laboratory in each dataset, the proximity between clusters decreases when simultaneous or permutation filtering is applied. This observation indicates that filtering decreases dissimilarity between samples that contain the same mock communities and slightly alleviates the effects of lab-to-lab variability. Thus, filtering achieves dimension reduction (reduces the number of taxa) while preserving beta diversity.

### 4.2. The Reagent and Laboratory Contamination Data

Figure 4C displays the difference in the Shannon index of the filtered outputs, corresponding to the *p*-value threshold 0.1, using simultaneous and permutation filtering among 6 dilution levels and 3 processing institutes. It is expected that as the dilution levels increase, more uncertainty in true taxa identification is introduced into the biological system, thus the proportion of signal taxa decreases whereas that of noise taxa increases. This phenomenon is displayed on the top heatmap in Figure 2A, where taxa are arranged from left to right in decreasing abundance order (noise taxa to the bottom right of the heatmap). As the dilution levels increase (rows of the heatmap), each dilution “band” becomes denser due to the increase in noise taxa counts. Dilution thus causes the true signal to become more, even with noise, and this consequently leads to a higher Shannon index. This effect may cause problems comparing alpha diversity for different groups of samples with variable biomass because it will be more difficult to differentiate between signal and noise taxa in low biomass samples. The filtering methods address this issue by removing noise taxa in highly diluted samples (dilutions 3, 4, and 5), where the simultaneous filtering removes more taxa than the permutation algorithm and has more impact on reducing the alpha diversity.

To compare the beta diversity for filtered outputs, the pairwise between-sample Bray–Curtis distances were calculated using the taxa matrices' combination with a similar set up to the analysis with the MBQC data. The PCoA ordination plot for the first two axes that explain 81.3% of the variability in the data is shown in Figure 4D. The six dilution levels and three filtering methods (none, simultaneous, and permutation PERFect) are arranged in columns and rows respectively; samples are colored according to the three processing institutes. Ideally, the samples from all three processing institutes should have the same composition of taxa regardless of the dilution levels. However, contaminants that went into the samples during the DNA extraand PCR process lead to the differences between the three processing institutes. Figure 4D shows that filtering does not dramatically change samples' pairwise distances in ordination plots. This is because PERFect, like many other filtering methods, removes taxa with low abundance which do not contribute to the signal, and thus do not dramatically affect samples' pairwise distances. These observations lead to the important conclusion that filtering reduces the number of taxa considered in the analysis, and thus reduces the dimensionality of the taxa table, without affecting beta diversity.

### 4.3. Alcoholic Hepatitis Data Analysis

#### 4.3.1. Random Forest Results

**Figure 5:**
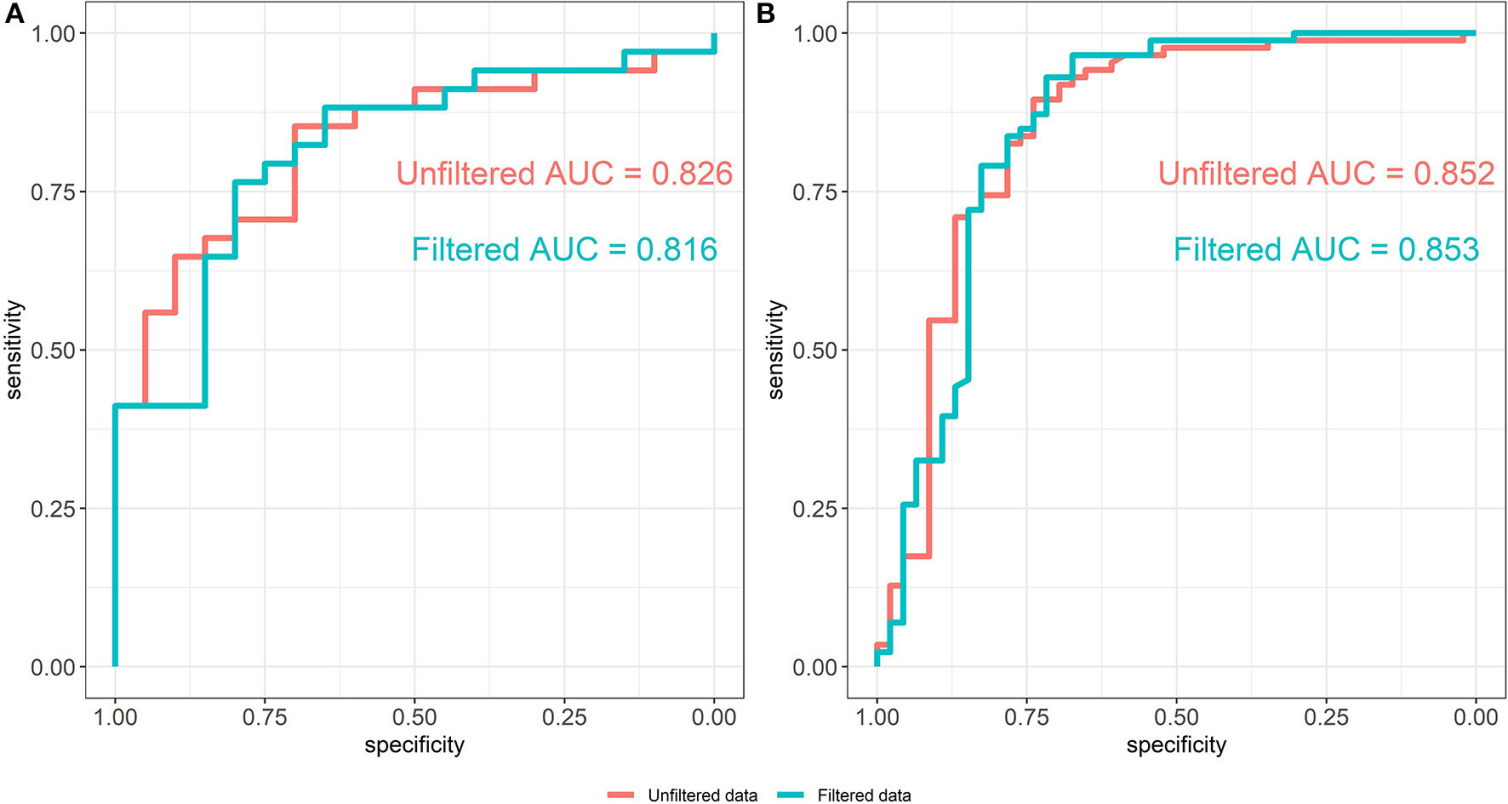
ROC curves of the random forest models from unfiltered and filtered data that are differentiated by colors. **(A)** ROC curves from the Alcoholic Hepatitis data (Smirnova et al., 2020). **(B)** ROC curves from the IBD data (Lloyd-Price et al., 2019).

The ROC curves of the random forest models between unfiltered (AUC = 0.826) and filtered (AUC = 0.816) data are shown in Figure 5A. The predictive abilities of the filtered and unfiltered models as measured by AUC values are similar, although there is a small decrease of 0.01 in the AUC for the model built on the filtered data. This implies that removing rare taxa has little effect on the classification ability of the random forest model. Further, the most predictive taxa, as measured by the mean Gini decrease criteria, also tend to be abundant (see Supplementary Table 1). Specifically, the ranks of the top 35 predictive taxa in the unfiltered data vary between the first and 81st most abundant taxa out of the total 345 taxa in the unfiltered data set.

To compare the discrepancy of the taxa importance rank between these two random forest models, we use the elbow method on the taxa mean Gini decrease in unfiltered data to choose the top 60 most predictive taxa. The taxa are chosen so that the differences of consecutive taxa mean Gini decrease are no less than 0.001. Then, their importance ranks are compared to those from the filtered data. Results indicate that in general, while there is minor variation, ranks are consistent and strong predictive taxa keep their classification ability after filtering (see Supplementary Table 1).

#### 4.3.2. LEfSe Results

**Figure 6:**
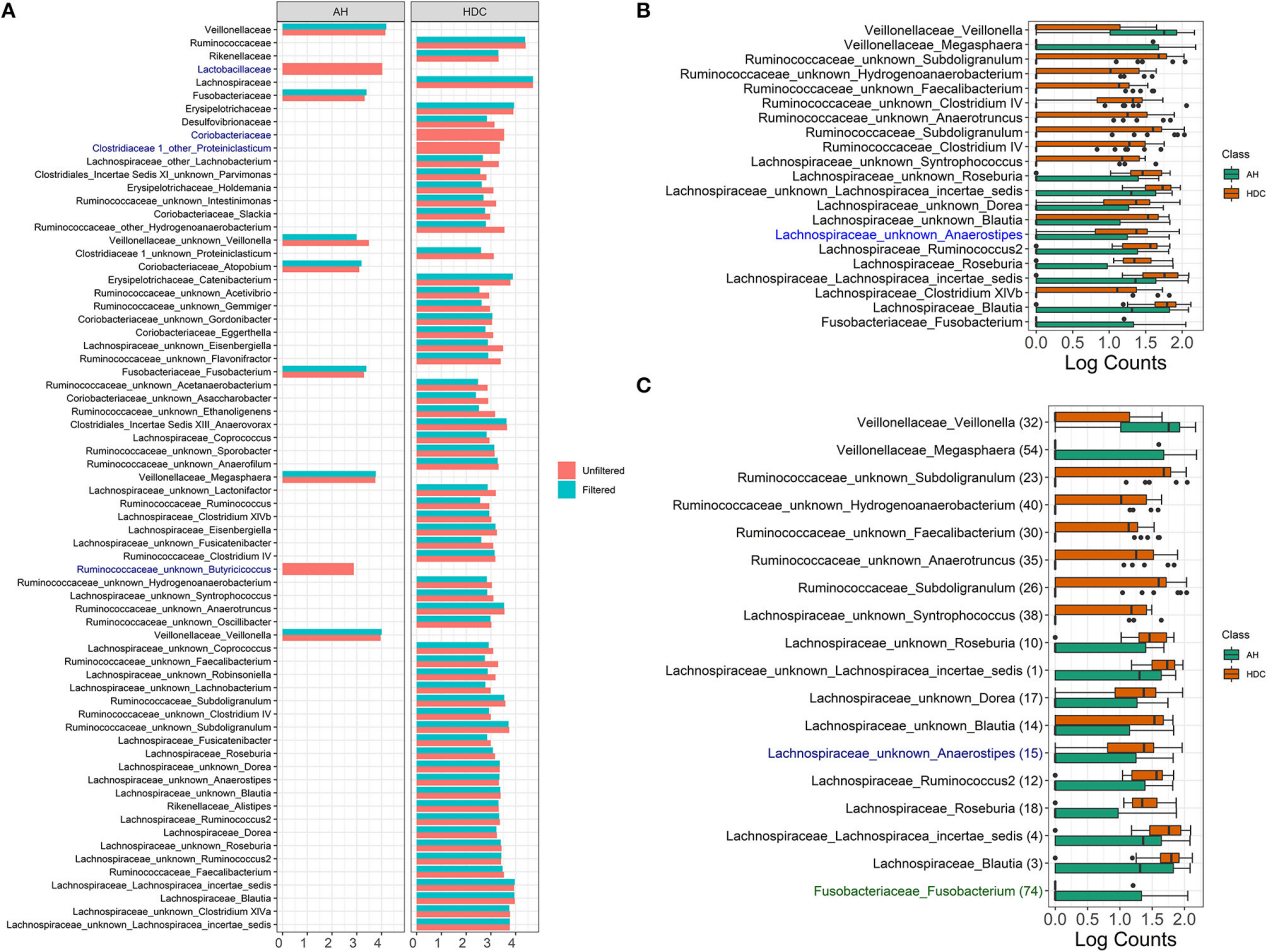
Alcoholic Hepatitis analysis results for Random Forest, LEfSe, and DESeq2. **(A)** Log fold changes for all significant taxa from LEfSe results from unfiltered and filtered data that are differentiated by colors. Taxa that are present in filtered data but are not significant are colored in dark blue. **(B)** Barchart of log(count+1) for significant taxa from DESeq2 results, colored by the disease states. Taxa that are present in filtered data but are not significant are colored in dark blue. **(C)** Barchart of log(count+1) for common significant taxa between random forest models, LEfSe, and DESeq2 results on unfiltered data, colored by the disease states. From filtered data, while black taxa are common results with those from unfiltered data, blue taxa are non-significant in DESeq2 results and green taxa are not in the top 60 predictive taxa in the random forest model. Data source: (Smirnova et al., 2020).

The LDA score for all significant taxa using LEfSe from unfiltered and filtered data are shown in Figure 6A. For each taxon, the log fold change values from unfiltered and filtered data are similar, although the values from unfiltered data tend to be slightly higher (range between 0.01 and 0.75). *This indicates that filtering retains the differential abundance for almost all taxa*, thus taxa that are significant in unfiltered data tend to be significant in filtered data.

There are four taxa that are present in the unfiltered but absent in the filtered data results, where two are identified at the family level (*Lactobacillaceae* and *Coriobacteriaceae*) and two are identified at the genus level (*Ruminococcaceae Butyricicoccus* and *Clostridiaceae Proteiniclasticum*). At the family level, filtering removes rare taxa from each family (3 out of 6 taxa from *Lactobacillaceae* and 6 out of 12 taxa from *Coriobacteriaceae*). The remaining taxa aggregated to each of the two families do not discriminate between the heavy drinking control (HDC, *n* = 20) and the Alcohol Hepatitis (AH, *n* = 34) group. At the genus level, *Ruminococcaceae Butyricicoccus* and *Clostridiaceae Proteiniclasticum* are flagged as significant in the unfiltered but as non-significant in the filtered data. Both taxa are overall rarer (42nd and 179th most abundant taxa out of 345 taxa), with relative abundance between 0 and 0.02 (max without outlier) (see Supplementary Figure 1), one outlier for *Ruminococcaceae Butyricicoccus* (relative abundance = 0.08), and only a few low relative abundance observations in the HDC group for *Ruminococcaceae Butyricicoccus*. This suggests that in the presence of taxa with outliers, the difference between groups appears to be stronger when tested in the unfiltered dataset with a large number of rare taxa. However, the strength of the outliers' effect is reduced when testing is performed in the filtered data, where extremely rare taxa are removed.

#### 4.3.3. DESeq2 Results

The DESeq2 method based on log(Count + 1) transformed data was used to identify differentially abundant taxa in filtered and unfiltered taxa tables; results are shown in Figure 6B. Taxa colored in black were identified as significant in both filtered and unfiltered datasets, while the taxon in blue (*Lachnospiraceae Anaerostipes*) was present in filtered data but was not significant. Specifically, at the alpha level of 0.1, this taxon was significant for both unfiltered and filtered data with a raw *p*-value of 0.028 and 0.035, respectively. After the *p*-value adjustment step using Benjamini–Hochberg procedure, it remained significant in unfiltered data (*p* = 0.093) but became non-significant in filtered data (*p* = 0.113). Since the change between raw *p*-values is relatively small and the adjusted *p*-values are close to the alpha level, we may conclude that for DESeq2 method, there are no major differences due to filtering in this dataset.

#### 4.3.4. Summary of Discrimination Results

Common significant taxa between random forest models LEfSe and DESeq2 results on unfiltered data are shown in Figure 6C. There are two taxa that are significant in the unfiltered but not significant in the filtered: *Lachnospiraceae Anaerostipes* (not significant if DESeq2) and *Fusobacteriaceae Fusobacterium* (low importance in the random forest). Results indicate that these discrepancies occur for the borderline significant taxa.

### 4.4. IBD Data Analysis

**Figure 7:**
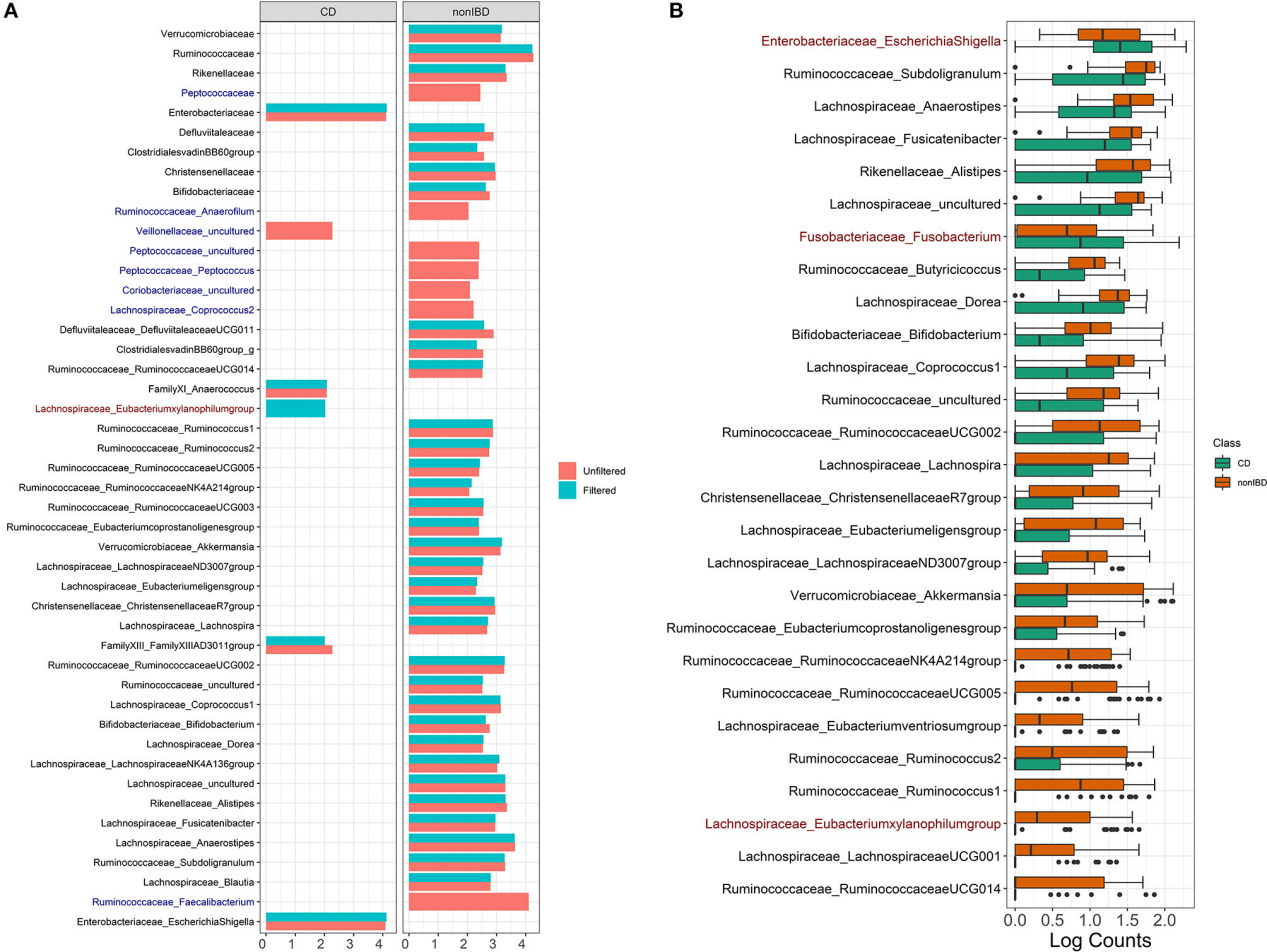
IBD analysis results for LEfSe and DESeq2. **(A)** Log fold changes for all significant taxa from LEfSe results from unfiltered and filtered data that are differentiated by colors. Taxa that are present in filtered data but are not significant are colored in dark blue. Taxa that are present in unfiltered data but are not significant are colored in dark red. **(B)** Barchart of log(count+1) for significant taxa from DESeq2 results colored by the disease states. Taxa that are present in unfiltered data but are not significant are colored in dark red. Data source: (Lloyd-Price et al., 2019).

Filtering analysis on the IBD data is performed using the workflow of Alcoholic Hepatitis data and the results are shown in Figures 5B,7. We observe similar results' patterns with the Alcoholic Hepatitis analyses: (1) significant taxa tend to be highly abundant; (2) predictive abilities of the filtered and unfiltered random forest models as measured by AUC values are similar (Figure 5B: unfiltered AUC = 0.852; filtered AUC = 0.853); (3) in the discriminant analysis, the differences between LDA scores for all significant taxa from unfiltered and filtered data are small; (4) the significance effect of more rare taxa with outliers is stronger in the unfiltered compared to filtered dataset.

Compared to Alcoholic Hepatitis data, DESeq2 identified three additional significant taxa in the filtered data: *Lachnospiraceae Eubacterium* (*p* = 0.080), *Fusobacteriaceae Fusobacterium* (*p* = 0.0999) and *Enterobacteriaceae EscherichiaShigella* (*p* = 0.070). Since these adjusted *p*-values are borderline significant in the filtered data that includes fewer taxa, these genera are non-significant when DESeq2 is run on the unfiltered data due to a larger number of taxa used in multiple comparison adjustment.

### 4.5. Comparison With Contaminant Removal Method

Contaminant removal and filtering methods have a common goal of identifying potential features derived due to technical limitations that occur with sequencing and taxonomic classification. However, the main goal of each method is different, which led to complementary effects in our comparison studies. Filtering concentrates on removing rare taxa relying mostly on sparsity assumptions and using no auxiliary information about the derived feature data. Contaminant removal methods implemented in R package decontam use additional information from the sequencing process to apply statistical threshold rules marginally to one taxon at a time. The decontam package implements two methods, each using specific auxiliary information about derived feature data: (1) the frequency method uses DNA quantitation data recording the concentration of DNA in each sample, and (2) the prevalence method employs a set of “negative control” samples in which sequencing was performed on blanks without any biological sample added. We applied decontam frequency method (Davis et al., 2018) to the reagent and laboratory contamination data to compare filtering and contaminant identification methods in terms of the type of taxa they remove and their effects on diversity. Results are illustrated in Figure 2, which compares the heatmaps, alpha, and beta diversity for the derived feature data without filtering the data where taxa are removed using decontam frequency and PERFect simultaneous filtering methods. Heatmaps in Figure 2A indicate that decontam frequency identifies abundant taxa as contaminants (left oval in the middle panel heatmap) leaving rare taxa to the right of the plot in the data set. In contrast, PERFect removes rare taxa (right oval in the bottom panel heatmap) that decontam was not able to detect, while leaving abundant taxa in the data set. These observations highlight important methodological differences between two methods. Specifically, decontam frequency fits a regression model to compare a contaminant model, in which expected frequency varies inversely with total DNA concentration, and a non-contaminant model, in which expected frequency is independent of total DNA concentration (Davis et al., 2018). For a rare taxa regression model fit is unstable due to the small number of observations (a few samples where rare taxa appear), and thus decontam returns missing values for the taxa significance. Specifically, out of a total of 635 taxa, decontam identified 61 taxa as contaminants and was not able to evaluate statistical significance for 221 taxa. This filtering approach has a major limitation of being skewed toward retaining more dominant features, as a result, a persistent contaminant feature might appear in a large number of samples, have a high contribution toward covariance, and would not be removed from the data set.

Comparison of alpha diversity in Figure 2B reveals that both decontam and PERFect reduce Shannon diversity. Based on this data, decontam leads to greater alpha diversity reduction, which is expected since it removes more abundant taxa. However, it should be noted that this is a small sample size study with only 18 observations (six observations per each of the three institutes), and the results may not be conclusive. Figure 2C compares beta diversity Bray–Curtis distance plots for each method (rows) by dilution level (columns) with samples colored by processing institutes. All samples contain the same biological material, thus under no technical variability scenario, the points should overlap on the plot. This is the case for undiluted samples (first column dilution = 0); however, the observed dissimilarity between samples increases with dilution. Davis et al. (2018) showed that removing abundant contaminants (second row in Figure 2C) reduces technical beta diversity. Comparing these results with filtering output confirms our previous observations based on the Microbiome Quality Control data set that removing rare taxa via filtering does not significantly effect beta diversity.

## 5. Conclusions

It is generally believed that filtering rare taxa is an effective quality control approach to remove possible contaminants, sequencing, and taxonomic assignment artifacts. The current study supports this paradigm and demonstrates that filtering has a strong potential to reduce lab-to-lab variability between samples that contain similar microbial species and are processed according to different protocols. Moreover, filtering removes rare taxa that have a low contribution to the signal, thus reducing the dimensionality of the data with minimal information loss. The ability of the methods to detect taxa significantly different across two disease groups is almost unaffected by filtering. Except for a small number of taxa detected as significant in unfiltered but not filtered (or visa versa) data, each method produces the same results. Major discrepancies in taxa that are identified as significant come from the data analysis method choice (Random Forest, LEfSe, or DESeq2) but not from filtering. To the best of our knowledge, *this is the first report entirely dedicated to the analysis of the effects of filtering rare taxa from the derived feature table on commonly used statistical analyses of microbiome data and detection of differentially abundant taxa in comparison of two disease groups*. The presented analysis is further facilitated by utilizing novel large microbiome quality control mock datasets and clinically relevant disease study datasets.

The statistical methodology literature on quality control for the derived feature data is scarce. Most previous studies either recommended filtering without a thorough evaluation of its effects (Goodrich et al., 2014; Cullen et al., 2020), or focused on the number of taxa removed from the mock artificial community studies (Smirnova et al., 2019) and on contaminant identification (Knights et al., 2011; Davis et al., 2018). We have previously demonstrated that filtering methods were effective in identifying true species in mock data (Smirnova et al., 2019). An underlying assumption of filtering is that most rare taxa are not informative in the analysis; however, the presence of rare taxa in the derived feature data increases sparsity and affects the performance of statistical methods. The current study supports earlier hypotheses and validates that removing rare taxa does not impact the scientific conclusions.

It has also been established that the contaminant removal method implemented in decontam package (Davis et al., 2018) was effective in reducing technical variability across processing institutes. Comparison of filtering and contaminant removal methods on the reagent and laboratory contamination data (Salter et al., 2014) reveals that the two methods have complementary effects: decontam removes persistent contaminant features that appear in a large number of samples while filtering removes rare taxa that appear in a small number of samples. This is not surprising because this is exactly the assumptions of these two methods; nevertheless, this is a significant finding which suggests that in practice both methods may be used to remove sequencing artifacts from the derived feature data.

Another noteworthy finding is that most significant taxa in unfiltered data were abundant. The random forest variable importance ranks of the top 35 predictive taxa in the unfiltered data ranged between (1) the 1st and 81st most abundant taxa out of the total 345 taxa in alcoholic hepatitis and (2) the 1st and 93rd most abundant taxa out of the total 409 taxa in the inflammatory bowel disease data set. Furthermore, in LEfSe and DESeq2 discrimination models, taxa that were found significant in filtered but not unfiltered data (or similarly in unfiltered but not filtered data) were overall more rare (present in small number of samples) and with low relative abundance. This is an important observation that may guide researchers' decisions regarding how aggressive filtering should be.

A limitation of filtering is that the reduction of type I errors (probability of removing important taxa) will inevitably increase type II errors (probability of keeping unimportant taxa). Indeed, if we want to be cautious in removing rare taxa to ensure that important taxa will remain in the data, we will not remove many taxa and will likely have a lot of unimportant taxa remaining; if we remove taxa aggressively, there is a high chance of filtering important rare taxa. In particular, in studies that aim to discover rare taxa, filtering would not be advisable since it will likely remove the rare but important taxa. This issue can be moderated by having a good understanding of the data (where the data are sampled and how they are generated) and using auxiliary study information that allows us to filter with confidence. In particular for predictive modeling, for example using a random forest approach in predicting alcoholic hepatitis, building a model with more abundant taxa may lead to higher reproducibility across studies as rare taxa may not be observed in another cohort sampled at different conditions. Another limitation of this study was the use of derived feature data that were obtained using an internal bioinformatics processing pipeline for each dataset. Future studies that start with the raw sequencing data and use the same bioinformatics pipeline may produce the evidence of the efficiency of filtering and contaminant removal methods.

We would like to stress that the goal of the current study is the evaluation of filtering methods on commonly used microbiome analyses. As a part of this study, filtering was compared to a closely related contaminant removal method implemented in R package decontam using a dataset that was previously illustrated by the package developers (Davis et al., 2018). It would be of interest to perform a thorough comparison of these methods on other datasets used in this study, however this is outside of the scope of this paper.

In summary, the current study provides information on the effects of removing rare taxa on technical variability and scientific conclusions drawn from statistical analyses. We provide insights into the role of filtering in microbiome studies, and highlight the importance of derived feature data quality control prior to scientific analysis.

## Data Availability Statement

The Microbiome Quality Control processed taxonomic features data was downloaded directly from the NIHHuman Microbiome Project Data Coordinating Center (HMP-DACC) (https://www.hmpdacc.org/MBQC/). The reagent and laboratory contamination dilution-series amplicon sequencing Samples for the S.bongori culture 16S rRNA gene profiling were deposited under European Nucleotide archive (ENA) project (https://www.ebi.ac.uk/ena/browser/guides) accession EMBL: ERP006737. Data processingsteps are described in scripts deposited at https://github.com/benjjneb/DecontamManuscript. Alcoholichepatitis data is generated as a part of the NIH TREAT consortium (https://www.treatcenter.org/about/) multi-center observational study (https://clinicaltrials.gov/ct2/show/NCT02172898). Inflammatory boweldisease data is a part of the Human Microbiome Project study led by the Broad Institute. The processed 16S taxonomic features data was downloaded directly from the Broad Institute website (https://ibdmdb.org/tunnel/public/summary.html). R version 3.6.0 was used with Bioconductor packagesphyloseq version 1.30.0, HMP2Data version 1.0.0, DESeq2 version 1.26.0, PERFect version 1.0.0. Analyses using LEfSe are done on the website https://huttenhower.sph.harvard.edu/galaxy/.

## Ethics Statement

The studies involving human participants were reviewed and approved by IRB. The patients/participants provided their written informed consent to participate in this study.

## Author Contributions

QC and ES designed the study. NC, KG, BK, VS, and AS collected and processed alcoholic hepatitis data. QC, XS, KR, AS, and ES analyzed the data. QC and ES drafted the manuscript. QC, XS, KR, NC, KG, BK, VS, AS, and ES reviewed the data and the manuscript. All authors contributed to the article and approved the submitted version.

## Conflict of Interest

The authors declare that the research was conducted in the absence of any commercial or financial relationships that could be construed as a potential conflict of interest.

## Abbreviations

AUC: area under the receiver operating characteristic curve
CD: Crohn's disease
HC: healthy control
HDC: heavy drinking control
HMP: Human Microbiome Project
IBD: inflammatory bowel disease
ICL: Imperial College London
LEfSe: linear discriminant analysis effect size
MAH: moderate alcoholic hepatitis
MBQC: microbiome quality control
PCoA: principal coordinates analysis
NGS: next-generation sequencing
PCR: polymerase chain reaction
rRNA: ribosomal RNA
SAH: severe alcoholic hepatitis
UB: University of Birmingham
WTSI: Wellcome Trust Sanger Institute.
