# Supplementary material for: Effects of Rare Microbiome Taxa Filtering on Statistical Analysis
Source: Front Microbiol. 2021 Jan 12;11:607325. doi: 10.3389/fmicb.2020.607325 (PMC7835481; doi:10.3389/fmicb.2020.607325)
Supplement: Supplementary file 1 [file Data_Sheet_1.pdf]

Supplementary Materials

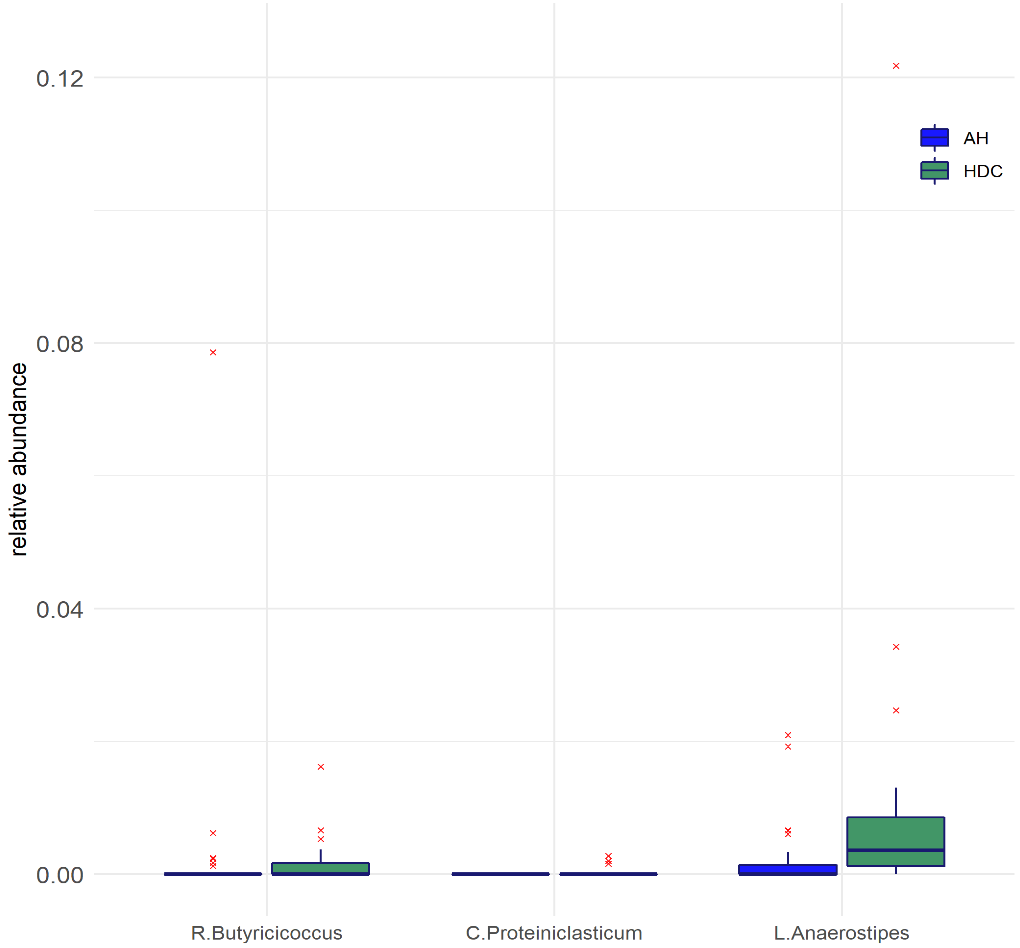

Figure 1: Alcoholic Hepatitis Data Analysis [1]. Taxa that are not present in the LEfSe and DESeq2 results from the filtered data when compared with the unfiltered data results. AH: Alcoholic Hepatitis group, HDC: Heavy Drinking Control group. Red crosses show relative abundance outliers.

Features	Mean			Number of samples present		Ranking		
	Decrease Gini	HDC	AH	HDC	AH	Abundance rank	RF Unfiltered rank	RF Filtered Rank
Veillonellaceae Veillonella	1.015	0.001	0.038	6	26	33	1	1
Ruminococcaceae unknown Anaerotruncus	0.737	0.012	0.001	14	5	36	2	4
Lachnospiraceae unknown Blautia	0.706	0.011	0.002	14	10	15	3	9
Lachnospiraceae unknown Lachnospiracea incertae sedis	0.705	0.029	0.006	20	23	2	4	7
Lachnospiraceae Roseburia	0.669	0.007	0.003	17	11	19	5	2
Lachnospiraceae unknown Syntrophococcus	0.658	0.002	0.000	12	3	39	6	6
Lachnospiraceae unknown Roseburia	0.646	0.011	0.002	17	16	11	7	3
Lachnospiraceae Clostridium.XlVb	0.598	0.003	0.001	11	3	48	8	17
Ruminococcaceae unknown Clostridium.IV	0.581	0.005	0.003	15	6	26	9	5
Lachnospiraceae unknown Lactonifactor	0.559	0.001	0.000	9	2	53	10	10
Ruminococcaceae unknown Subdoligranulum	0.519	0.024	0.005	13	6	24	11	13
Lachnospiraceae unknown Coprococcus	0.429	0.005	0.002	12	6	32	12	12
Lachnospiraceae unknown Anaerostipes	0.414	0.012	0.002	15	10	16	13	14
Ruminococcaceae Clostridium.IV	0.393	0.005	0.001	13	7	43	14	24
Ruminococcaceae unknown Faecalibacterium	0.391	0.002	0.001	13	5	31	15	11
Lachnospiraceae unknown Ruminococcus2	0.387	0.015	0.004	16	15	10	16	18
Lachnospiraceae Ruminococcus2	0.379	0.010	0.003	17	12	13	17	26
Lachnospiraceae Fusicatenibacter	0.378	0.005	0.003	14	9	20	18	21
Lachnospiraceae Blautia	0.376	0.048	0.014	19	21	4	19	8
Streptococcaceae Streptococcus	0.369	0.055	0.056	17	31	8	20	29
Lachnospiraceae unknown Clostridium.XlVa	0.358	0.027	0.006	20	25	3	21	28
Ruminococcaceae unknown Anaerofilum	0.355	0.007	0.001	10	6	56	22	23
Ruminococcaceae unknown Acetanaerobacterium	0.346	0.001	0.000	8	1	67	23	37
Rikenellaceae Alistipes	0.331	0.013	0.003	14	13	14	24	22
Lachnospiraceae unknown Dorea	0.331	0.010	0.001	15	11	18	25	27
Porphyromonadaceae Parabacteroides	0.324	0.014	0.036	19	20	7	26	25
Lachnospiraceae Lachnospiracea incertae sedis	0.320	0.052	0.014	19	21	5	27	31
Lachnospiraceae Eisenbergiella	0.286	0.007	0.002	11	11	47	28	32
Bacteroidaceae Bacteroides	0.277	0.204	0.233	19	29	1	29	16
Ruminococcaceae Subdoligranulum	0.269	0.019	0.006	13	6	27	30	15
Ruminococcaceae unknown Oscillibacter	0.264	0.004	0.000	9	3	35	31	34
Porphyromonadaceae Odoribacter	0.255	0.003	0.000	3	3	68	32	35
Ruminococcaceae Oscillibacter	0.243	0.002	0.001	8	5	29	33	41
Lachnospiraceae Coprococcus	0.232	0.003	0.001	8	2	59	34	33
Lachnospiraceae unknown Eisenbergiella	0.216	0.001	0.000	7	1	81	35	51

Table 1: Alcoholic Hepatitis Data Analysis [1]. Top 35 predictive taxa in the final random forest model using unfiltered data. AH: Alcoholic Hepatitis, HDC: Heavy Drinking Control. These taxa are consistently abundant, indicated by their abundance rank, which is defined by the number of samples a taxon is present in. There is also a strong agreement between the random forest (RF) variable importance rank (according to Mean Decrease Gini index) in unfiltered and filtered data.

	Mean			Number of samples present		Ranking		
Features	Decrease Gini	non IBD	CD	non IBD	CD	Abundance rank	RF Unfiltered rank	RF Filtered Rank
Lachnospiraceae LachnospiraceaeND3007group	2.330	0.002	0.000	37	27	47	1	1
Bifidobacteriaceae Bifidobacterium	1.898	0.003	0.002	41	49	27	2	2
Ruminococcaceae Ruminococcus1	1.428	0.003	0.001	29	16	66	3	4
Lachnospiraceae Eubacteriumeligensgroup	1.329	0.002	0.002	34	30	46	4	6
Ruminococcaceae RuminococcaceaeUCG002	1.245	0.010	0.004	37	42	34	5	5
Lachnospiraceae uncultured	1.104	0.013	0.006	42	59	19	6	9
Ruminococcaceae Subdoligranulum	1.086	0.022	0.014	42	68	11	7	12
Christensenellaceae ChristensenellaceaeR7group	1.019	0.005	0.002	35	30	45	8	11
Lachnospiraceae LachnospiraceaeUCG001	0.998	0.001	0.000	25	11	78	9	8
Ruminococcaceae Butyricicoccus	0.978	0.001	0.001	39	52	23	10	3
Lachnospiraceae Fusicatenibacter	0.937	0.009	0.005	43	61	13	11	16
Lachnospiraceae Anaerostipes	0.935	0.024	0.009	42	68	12	12	7
Lachnospiraceae Eubacteriumventriosumgroup	0.795	0.001	0.000	28	20	63	13	24
Lachnospiraceae Dorea	0.777	0.004	0.003	41	50	26	14	13
Enterobacteriaceae EscherichiaShigella	0.751	0.036	0.078	46	84	1	15	10
Lachnospiraceae Lachnospira	0.747	0.004	0.002	32	34	44	16	15
Bacteroidaceae Bacteroides	0.732	0.309	0.285	45	85	2	17	14
Ruminococcaceae RuminococcaceaeNK4A214group	0.728	0.001	0.001	29	21	60	18	17
Verrucomicrobiaceae Akkermansia	0.716	0.013	0.010	29	31	49	19	31
Ruminococcaceae RuminococcaceaeUCG014	0.688	0.002	0.001	17	10	93	20	19
Ruminococcaceae RuminococcaceaeUCG005	0.654	0.003	0.002	28	21	62	21	41
Fusobacteriaceae Fusobacterium	0.650	0.003	0.021	34	59	21	22	23
Lachnospiraceae Coprococcus1	0.598	0.008	0.004	39	46	30	23	25
Lachnospiraceae Blautia	0.579	0.013	0.012	42	71	10	24	55
Lachnospiraceae Lachnoclostridium	0.569	0.020	0.030	41	75	7	25	30
Erysipelotrichaceae Clostridiuminnocuumgroup	0.568	0.003	0.001	17	39	52	26	35
Ruminococcaceae Faecalibacterium	0.561	0.162	0.120	45	79	3	27	48
Ruminococcaceae Ruminococcus2	0.556	0.003	0.001	25	23	64	28	27
Rikenellaceae Alistipes	0.539	0.022	0.013	41	61	17	29	29
Veillonellaceae Veillonella	0.530	0.008	0.010	44	70	9	30	20
Lachnospiraceae Eubacteriumxylanophilumgroup	0.528	0.001	0.001	23	17	73	31	22
Lachnospiraceae LachnospiraceaeUCG008	0.523	0.015	0.013	42	79	6	32	36
Erysipelotrichaceae Holdemania	0.522	0.000	0.000	33	36	39	33	38
Erysipelotrichaceae Erysipelatoclostridium	0.499	0.002	0.001	28	43	38	34	32
Ruminococcaceae Eubacteriumcoprostanoligenesgroup	0.496	0.001	0.001	27	26	56	35	69

Table 2: IBD Data Analysis [2]. Top 35 predictive taxa in the final random forest model using unfiltered data. Non IBD: patients without Inflammatory Bowel Disease and CD: patients with Crohn's Disease. These taxa are consistently abundant, indicated by their abundance rank, which is defined by the number of samples a taxon is present in. There is also a strong agreement between the random forest (RF) variable importance rank (according to Mean Decrease Gini index) in unfiltered and filtered data.

References

[1] Smirnova, E., Puri, P., Muthiah, M.D., Daitya, K., Brown, R., Chalasani, N., Liangpunsakul, S., Shah, V.H., Gelow, K., Siddiqui, M.S., et al.: Fecal microbiome distinguishes alcohol consumption from alcoholic hepatitis but does not discriminate disease severity. Hepatology (2020)

[2] Lloyd-Price, J., Arze, C., Ananthakrishnan, A.N., Schirmer, M., Avila-Pacheco, J., Poon, T.W., Andrews, E., Ajami, N.J., Bonham, K.S., Brislawn, C.J., *et al.*: Multi-omics of the gut microbial ecosystem in inflammatory bowel diseases. Nature **569**(7758), 655–662 (2019)
